# Lymph Node Subcapsular Sinus Macrophages as the Frontline of Lymphatic Immune Defense

**DOI:** 10.3389/fimmu.2019.00347

**Published:** 2019-02-28

**Authors:** Dante Alexander Patrick Louie, Shan Liao

**Affiliations:** Department of Microbiology, Immunology and Infectious Diseases, The Snyder Institute for Chronic Diseases, Cumming School of Medicine, University of Calgary, Calgary, AB, Canada

**Keywords:** subcapsular sinus macrophage, CD169, lymph node (LN), free-floating antigens, virus, bacteria, cancer

## Abstract

Lymphatic vessels collect and transport lymph and pathogens to the draining lymph node (LN) to generate proper immune protection. A layer of macrophages that strategically line the LN subcapsular sinus (SCS) is directly exposed to the afferent lymph and are denoted as SCS macrophages. These macrophages are the frontline of immune defense that interact with lymph-borne antigens. The importance of these macrophages in limiting the spread of pathogens has been demonstrated in both viral and bacterial infection. In anti-microbial responses, these macrophages can directly or indirectly activate other LN innate immune cells to fight against pathogens, as well as activate T cells or B cells for adaptive immunity. As the first layer of immune cells embracing the tumor-derived antigens, SCS macrophages also actively participate in cancer immune regulation. Recent studies have shown that the LNs' SCS macrophage layer is interrupted in disease models. Despite their importance in fighting the spread of pathogens and in activating anti-tumor immunity, the mechanism and the immunological functional consequences for their disruption are not well-understood. Understanding the mechanism of these macrophages will enhance their capability for therapeutic targeting.

## Introduction

The lymphatic system consists of two major parts: lymphatic vessels and lymph nodes (LNs). Lymphatic vessels are present throughout the body, acting as a road map for immune surveillance. These vessels are responsible for collecting interstitial fluid, soluble proteins, peptides, metabolites, invading pathogens, and immune cells in the tissue, then transporting the collected contents to the draining LNs via afferent lymphatic vessels (1–3). Initial lymphatic vessels (also named lymphatic capillaries) have discontinuous junction molecules which are highly permeable, and permit easy access of fluid and other content from peripheral tissues (4). Initial lymphatic vessels congregate to contractile lymphatic vessels, also known as collecting lymphatic vessels. Collecting lymphatic vessels direct lymph to the LN. Once in the LN, free-floating antigens, migrating antigen-presenting cells, and resident LN immune cells meet to initiate immune activation. After immune surveillance in the LN, efferent lymphatic vessels return lymph and activated immune cells to the circulation in order to enter the site of pathogen invasion for immune protection.

**Figure 1:**
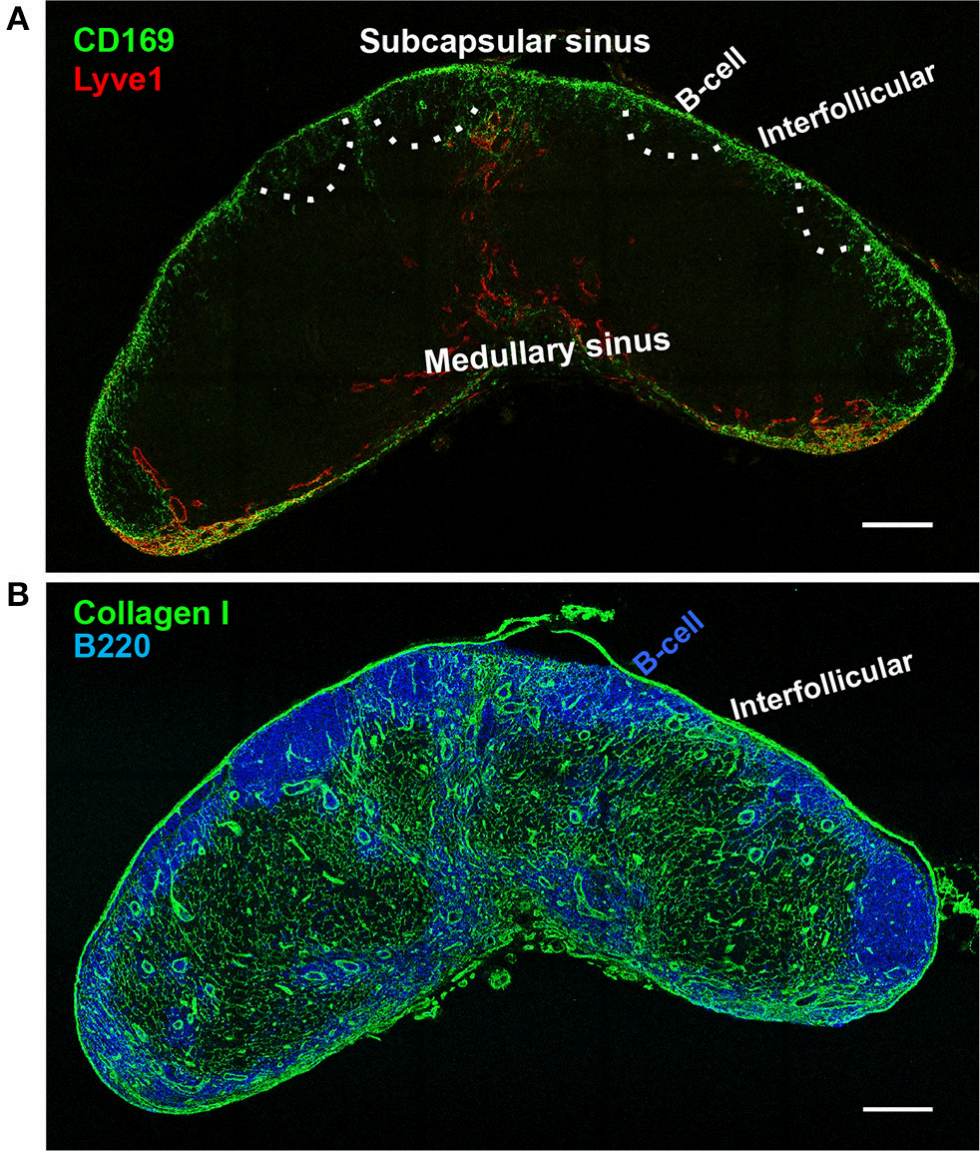
Lymph node sinus macrophages. Confocal microscope image of a wild-type inguinal lymph node at 20 × magnification. **(A)** Lymph node subcapsular sinus (SCS) and the medullary sinus (MS) are distinguished by the morphology of lymphatic endothelial cells (Lyve-1, red). CD169^+^ macrophages are concentrated in the SCS, with much sparser distribution in the MS (CD169, green). B-cell zones are indicated by dashed lines according to the staining using serial section in **(B)**. **(B)** Underneath the SCS macrophages are the B cell follicles (B220). Between the B cell follicles are the interfollicular zones which contain collagen I^+^ conduits. SCS macrophages are restricted in the SCS, but invade slightly deeper into the LN parenchyma at the interfollicular zone (Collagen I, green). Scale bars, 200 μm.

The transport of tissue-originated antigen-loaded antigen-presenting cells via lymphatic vessels has been largely studied. Migrating dendritic cells enter lymphatic vessels through the portals formed by the discontinuous basement membrane between adjacent endothelial cells, and is dependent on CCR7 expression on dendritic cells (DC) and chemokines CCL19 and CC21 expressed on lymphatic endothelial cells (5–8). However, not all antigens transported in lymphatics are loaded on dendritic cells. Some free-floating lymph-borne antigens can travel with lymph to the LN. The importance of how LN-resident antigen-presenting cells react to free-floating antigens in lymph has been gaining more interest in the past decade. As lymph enters the LN, fluid fills the sinus lumen. Lining the floor of the sinuses are sinus macrophages that directly embrace the lymph coming from the afferent lymphatic vessels (Figure 1). These macrophages sample the free-floating antigens in the afferent lymph within several minutes after administration of model antigen tracers or pathogens (9, 10). Larger molecules and particles, such as viruses and bacteria, are captured by sinus macrophages (11–15) and sinus DCs (16). Smaller antigens, such as ovalbumin (OVA), can be captured by sinus macrophages and DCs. Additionally, smaller antigens can enter the LN conduits and are sampled by the LN conduit-associated DCs (17, 18). The first wave of DC activation occurs several hours before tissue-originated antigen-bearing DCs enter the LN, acting as another layer of protection in the event pathogens evade detection at the site of invasion (18–21). In fact, even in the absence of tissue-migrating antigen-presenting cells, the LN-resident antigen-presenting cells are capable of generating a protective immune response against invading pathogens (16, 22, 23). Therefore, LN sinus resident macrophages function as a frontline of immune protection to lymph-borne pathogens.

During cancer lymphatic metastasis, metastatic tumor cells and tumor-derived antigens travel through lymphatic vessels to the tumor draining lymph node. Metastatic tumor cells were observed to first accumulate at the subcapsular sinus (24). LN metastatic tumor cells can invade the LN blood vessels as early as 2 days post-injection and spread to distant organs from the tumor draining LN (25, 26). Subcapsular sinus macrophages are the first layer of immune cells that are exposed to the metastatic tumor cells and tumor-derived antigens coming from the afferent lymphatic vessels. Studies in this field can reveal exciting new prospects when it comes to developing cancer immunotherapy. We reviewed the literature on how these macrophages are responsible for activating an immune response to the invading pathogens or tumor-derived antigens, as well as how the interruption of these macrophages in the LN is associated with disease.

## LN Sinus Macrophages

Sinus macrophages are not uniform across the entire LN; they can be subdivided into two major populations: the subcapsular sinus (SCS) macrophages and the medullary sinus macrophages according to their anatomical location in the LN (Figure 1). There are also sinus dendritic cells that sparsely populate the subcapsular sinus. Functionally, both sinus macrophages and DCs can acquire pathogen or particles from the passing lymph in the SCS. The sinus macrophages differ phenotypically, as SCS macrophages express Mac1 (CD11b/CD18), Siglec-1 (CD169), but lack the expression of F4/80, a murine macrophage marker (27). On top of that, a small proportion of the SCS CD169^+^ cells are CD169^+^CD11c^+^, indicating their DC phenotype (12, 16). Yet researchers still title these cells as macrophages, because, despite lacking the common F4/80 murine macrophage marker, SCS macrophage differentiation depends on the “macrophage colony-stimulating factor” cytokine, also known as CSF-1 (27–29). On the other hand, the phenotype of medullary sinus macrophages is more indicative of their macrophage characterization as they express F4/80 and Mac1. Some of the medullary sinus macrophages also express CD169 at a relatively lower level, therefore SCS macrophages are specifically distinguished as CD169^+^F4/80^−^, while medullary sinus macrophages are CD169^+^F4/80^+^ or CD169^low/−^F4/80^+^ (9).

Sinus macrophages also differ from each other functionally. Classically activated macrophages, known as M1 macrophages, typically produce pro-inflammatory cytokines, mediate pathogen resistance, and contribute to tissue destruction (30). This largely describes the medullary sinus macrophages, given their high lysozyme content and ability to process antigens, but no evidence has been shown for their capability to produce pro-inflammatory cytokines (31, 32). In contrast, SCS macrophages show relatively low phagocytic activity, but have demonstrated the ability to produce pro-inflammatory cytokines, namely type I interferon's (27, 33, 34). Therefore, while both sinus macrophages exhibit components of M1 macrophage function, a consensus on their categorization has yet to be reached in the field.

The origin and development of SCS macrophages has been studied to better understand their function. As stated earlier, the CSF-1/CSF-1 receptor signaling interaction is pivotal for the presence of SCS macrophages. Transgenic mice with a recessive osteopetrotic mutation (op/op) demonstrate a CSF-1 deficiency and show a significant reduction in SCS macrophages. Similarly, anti-CSF-1 receptor treatment to block the CSF-1 ligand from binding to CSF-1 receptor significantly depleted SCS macrophages, while medullary sinus macrophages remained intact (28). However, while medullary sinus macrophages are unaffected by blocking CSF-1/CSF-1 receptor interaction, CSF-1 receptor deficient mice show a significant depletion of F4/80^+^ macrophages, indicating the requirement of CSF-1 receptor activation for F4/80^+^ macrophage development (29). In addition to CSF-1, SCS macrophages appear to need the lymphotoxin signal for their development. Lymphotoxin receptor LTβR is shown to be present on the surface of both SCS macrophages and medullary sinus macrophages, however chimeric mice lacking the LTβR (*ltbr*^−/−^*)* only show a deficiency in SCS macrophages (27). The activation of LTβR on SCS macrophages largely depends on LTα_1_β_2_, the ligand for LTβR, present on LN B cells that are located just underneath the SCS in the LN. μMT mice, which lack mature B cells in the LN, show significantly fewer macrophages with the SCS phenotype (CD169^+^F4/80^−^) and an abundance of the medullary sinus phenotype (CD169^+^F4/80^+^) (34). Furthermore, by ablating lymphotoxin signaling with LTβR-Ig, a soluble lymphotoxin receptor that blocks downstream signaling, a similar deficiency in the SCS macrophage phenotype can be found in wild-type mice as the μMT mice. Medullary sinus macrophages appeared unaffected by lymphotoxin signaling blockade (34). Based on these observations, while medullary sinus macrophages rely on CSF-1 receptor signaling for their development, SCS macrophages require CSF-1 receptor and LTβR for their development and the maintenance of their phenotype.

## SCS Macrophages Prevent Lymph-Borne Pathogen Systemic Spreading

**Figure 2:**
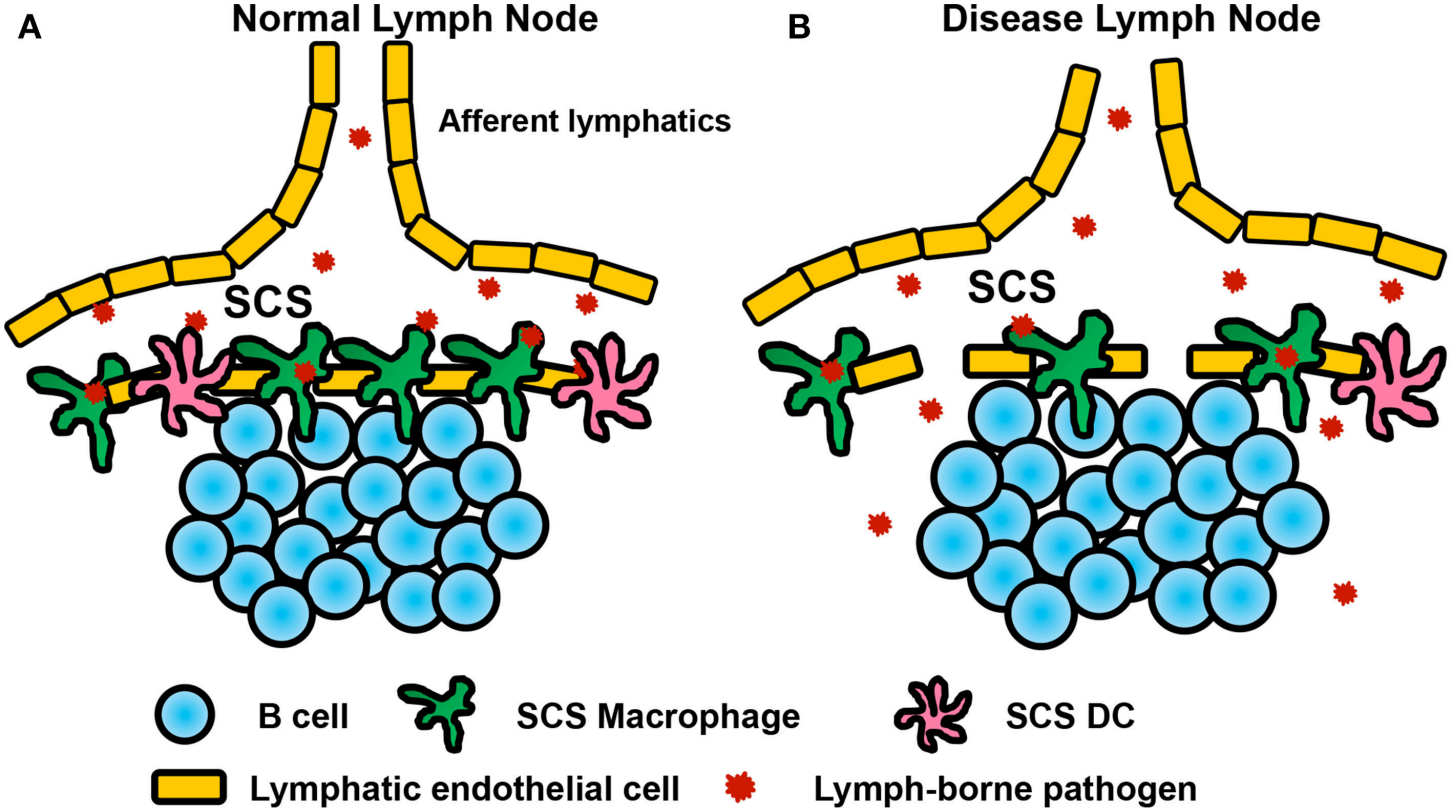
Function of the subcapsular sinus macrophage layer in normal and inflamed lymph nodes. **(A)** Lymph-borne free floating particles and pathogens travel with lymph and enter the lymph node subcapsular sinus via the afferent lymphatics. Subcapsular sinus macrophages are the first layer of cells in the draining lymph node that capture and retain lymph-borne pathogens from entering the lymph node parenchyma likely via the interaction between CD169 and its ligand, α2,3-linked sialic acids, expressed on the surface of cells or microbes. After pathogen capture, SCS macrophages can relay the antigen to B cells just underneath the SCS to prime B cell and humoral responses. SCS macrophage activation produces different types of cytokines to recruit and communicate with other immune cells, such as NK cells, γδ T cells, non-classical CD8^+^ T cells, neutrophils, monocytes, T cells etc. to combat the invading pathogens. The SCS macrophage layer prevents pathogen from invading the lymph node parenchyma or systemic spreading. **(B)** In an inflamed LN during diseased condition, the SCS macrophage layer is interrupted, allowing pathogen to invade the lymph node parenchyma or systemic spreading. The immunological consequence of disrupting SCS macrophage appears contraversial in different types of infection or in cancer progression. The reason behind SCS macrophage layer disruption remains unclear as well.

Because SCS macrophages directly embrace pathogenic particles arriving from afferent lymphatic vessels, SCS macrophages have been widely studied in antimicrobial immunity, including anti-viral and anti-bacterial responses (Figure 2A). Studies on the function of SCS macrophages has first been demonstrated in preventing virus from spreading from the LN to the blood circulation or other organs after subcutaneous infection. Multiphoton intravital microscopy showed CD11b^+^CD169^+^MHCII^+^ macrophages located on the floor of the popliteal SCS functioning as a “flypaper” to capture fluorescently labeled vesicular stomatitis virus (VSV) particles after a subcutaneous injection at the footpad (11). This observation extends to different viruses, such adenovirus, vaccinia virus and murine cytomegalovirus (MCMV), as luciferase-labeled MCMV is limited to the LN for several days before spreading systemically (11, 35). Artificially depleting the SCS macrophages prior to VSV challenge led to a significant reduction in animal survival and a marked increase in viral titers found in the brain and spinal cord (33).

The “flypaper” function of SCS macrophages is also applicable to lymph-borne bacteria. Fluorescently labeled *Pseudomonas aeruginosa*, an extracellular bacterium, was found in the LN parenchyma and blood 8 h post-injection when the macrophages were depleted, while bacteria were limited to the SCS when the macrophage layer was intact (36). More specifically, lipid antigens, such as lipopolysaccharide found on bacteria, has also been shown to localize with the SCS macrophages (37). This further defines the “flypaper” function of the SCS macrophages as it is not only preventing a systemic spread, but specifically limits pathogens to the SCS in the LN. Restricting pathogens to the SCS is at least partially achieved by the expression of CD169 on the macrophages, as CD169 interacts with α2,3-linked sialic acids expressed on the surface of cells or microbes. Biotinylated exosomes specifically bound to SCS macrophages on tissue sections while biotinylated bovine serum did not, suggesting the CD169^+^ macrophages retain extracellular vesicles and microbes rather than free flow proteins at the sinus (38).

However, evident by their minimal phagocytosis function and failure to adequately process the self-quenching DQ Green probe, SCS macrophages are poorly phagocytic and cannot clear the microbes directly (27). Instead, these macrophages ensure enough immune stimulation by supporting replication of captured pathogens. Fluorescently labeled VSV was robustly replicated in wild-type LNs, while mice lacking the SCS macrophages showed no virus replication (33, 34). Without the immune protection generated by this layer of macrophages, viruses may invade deeper into the parenchyma and infect LN neurons or LN fibroblasts, and eventually disseminate into other organs (33, 35). The mechanism of SCS restricting virus spreading does not apply to all types of viral infection. Capture of the influenza virus alternatively depends on medullary sinus DCs to generate durable B cell responses (39). Like the subcapsular sinus macrophages, medullary sinus macrophages recruit additional immune cells to clear their targeted pathogen. While both SCS macrophages and medullary sinus macrophages demonstrate early activation marker CD69 to UV-inactivated influenza virus, medullary sinus macrophages have been shown to be preferentially activated through the secretion of IFN-β, which further induces IL-1α expression, leading to the expression of dendritic cell and monocyte chemoattractant, MCP-1 (40). Understanding the mechanism of how influenza virus escape SCS macrophages and alternatively activate medullary sinus macrophages may help influenza vaccine design.

Once activated, SCS macrophages function by communicating with other LN resident lymphocytes or recruiting other cells to the SCS to provide rapid and robust anti-microbial responses to lymph-borne antigens. As B cells reside directly underneath the SCS macrophage layer, the early studies exploring the function of SCS macrophages identified that activating these cells attract B cells from the follicles to the SCS. SCS macrophages then relay captured antigens to B cells using a complement-dependent and -independent pathway (41). Multiphoton intravital microscopy visualized the accumulation of virus serotype-specific B cells at the SCS depending on the virus challenge, indicating their migration is highly selective (11, 42). Co-stimulatory molecule CD86 was upregulated and B cell receptors were internalized within 6 h after virus challenge, indicative of the activation of B cells. B cell activation after viral challenge failed in the LN when SCS macrophages were depleted with clodronate liposome (CLL). After the early activation, B cells migrate to the boundary between the B and T cell zones of the LN (43). Here, an interaction occurs between the primed B cell and the helper T cells, causing a proliferation of B cells and germinal center formation. Upon macrophage depletion, the antiviral B cells remained spread in the LN and take a much longer time to migrate to T-B cell border. However, depleting the macrophage layer only delayed rather than completely prevented B cell activation or humoral responses, raising the question about the exact function of these cells (11).

Antigen challenge also recruits innate immune cells to the LN SCS. In the case of lipid antigens, α-galactosylceramide was used to coat 200 nm silica particles to stimulate immune cell activation. iNKT cells migrate toward the SCS and are arrested within a few hours. Three days post-injection, the LNs had been inflamed and the number of iNKT cells present in the LN were 10-fold higher than normal (37). Modified vaccinia virus Ankara, a viral vector, was shown to induce NK cell motility and transition from the interfollicular zone and outer T cell zone to the SCS. Depleting the SCS macrophages with CLL reduced the NK cell accumulation and activation normally triggered by virus challenge (44). Similar to the viral challenge, parasite infection with *Toxoplasma gondii* causes NK cell accumulation in the SCS. However, depletion of SCS macrophage with CLL did not reduce the proportion of NK cells, only suppressed NK cell activation during *T. gondii* infection (45). Parasitic challenges such as QS-21, an adjuvant component of malaria, colocalized with SCS macrophages. Depleting these macrophages using CLL reduced monocyte, neutrophil, and dendritic cell recruitment to the draining LN (46). However, while neutrophil recruitment to the LN occurred in response to *Staphylococcus aureus* infection, SCS macrophage depletion via CLL did not change neutrophil movement to SCS (47). Based on these results, it is apparent that SCS macrophage activation recruits and activates different types of immune cells to the SCS when responding to different types of lymph-borne microbes.

SCS macrophages appear to produce different types of cytokines to aid in their function, which potentially explains the different immune cell recruitment against lymph-borne pathogens. In response to lymph-borne virus pathogens, infected SCS macrophages produce interferon-α. Activated SCS macrophages additionally recruit plasmacytoid DCs to the SCS to express type I interferon to initiate anti-viral immunity (33). Mice lacking the SCS macrophages show lower levels of both interferon-α and interferon-β mRNA levels in their LNs, which is correlated with a lower survival rate after viral challenge (34). In the case of bacterial infection, the number of interferon-γ producing lymphoid cells increased 4 h after subcutaneous *P. aeruginosa* infection (36). SCS macrophages are not necessarily the only cell population to express interferon-γ, as the NLR dependent inflammasome activation in these cells enhances other lymphoid cells, such as NK cells, γδ T cells, and non-classical CD8^+^ T cells, to produce interferon-γ. Absence of the SCS macrophages significantly reduces cytokine production and limits the recruitment of important innate immune cells to restrict bacteria spread (36). This could also adversely affect the initiation of the adaptive immunity as inflammasome activation induces an influx of innate immune cells and T cells (48).

## Interruption of SCS Macrophages in Diseases

While the early activation of SCS macrophages has been studied extensively, disease models pose a different perspective. It appears that pathogen-induced inflammation can disrupt the SCS macrophage layer (Figure 2B). Recruitment of neutrophils or NK cells appear to interrupt the SCS macrophage layer, but the mechanism and the function of SCS macrophage dissociation from the SCS remains unclear (45, 49). Using either CpG or LPS, the SCS macrophages were able to dissociate from the SCS, leading to disrupted protective layer. The observed macrophage dissociation appears to be CCR7 dependent, as CCR7-deficient animals seem resistant to inflammation-induced SCS macrophage disruption. Transferring activated bone marrow derived DCs to the LN is sufficient to disrupt SCS macrophage layer, indicating DC activation may cause SCS macrophages to dissociate from the SCS. However, since a subpopulation of CD169^+^ macrophages are CD11c^+^, whether the CCR7-dependent SCS macrophage dissociation is only restricted to CD169^+^CD11c^+^ cell or all SCS CD169^+^ cells remains unclear. The mechanism of SCS macrophage dissociation from the SCS remains to be clarified. When SCS macrophages were dissociated during inflammation, B cells were incapable of receiving the antigen and showed diminished activation as measured by germinal center formation and immunoglobulin production (50). These results appear to contradict experimental SCS macrophage depletion, where only early cognate B cell migration to SCS or to the border of T cell and B cell zone is affected, but does not prevent total B cell activation (11, 15). Influenza vaccination is capable of inducing lymph node subcapsular sinus and medullary sinus macrophage necrosis 12 h post-injection. However, the necrosis was independent of neutrophil or NK cell recruitment. Virus challenge activated TLR7 and Myd88, causing necrosis of the subcapsular sinus, but not the medullary sinus, macrophages (40).

Recent interest has sparked over these macrophages in the context of anti-tumor immunity. LN metastases are a key component in patient prognosis. Metastatic tumor cells present in the sentinel lymph node were able to spread systemically via the lymph node blood vessels or the efferent lymphatic vessels (25, 26). SCS macrophages in the tumor draining LN directly interacts with metastatic tumor cells or tumor-derived antigens coming from the afferent lymphatic vessels. The idea that SCS macrophages can limit the spread of cancer, similar to how they limit the spread of lymph-borne microbes, has developed into a relatively new field of study. Clinical studies have determined a correlation between CD169^+^ macrophage density in human sentinel LNs and a favorable tumor prognosis. Consistent between multiple different types of tumors, indicators for the favorable prognosis often include a lower number of LN metastases and increased CD8^+^ T cell tumor infiltration, reflecting the SCS macrophages' functions of limiting cancer spread and immune activation (51–53). To activate the antitumor CD8^+^ T cell response, SCS macrophages are capable of capturing irradiated tumor cells. Like microbes, subcutaneously injected apoptotic tumor cells travel to the LN and are captured mainly by CD169^+^ macrophages. Then, activated SCS macrophages recruit and prime anti-tumor CD8^+^ T cells at the SCS. Mice with their CD169^+^ cells depleted in a CD169-DTR model were incapable of activating CD8^+^ T cells or rejecting tumor cells after a vaccination with irradiated tumor cells (12).

However, a growing tumor and its complex tumor microenvironment significantly changes the function of SCS macrophages. Instead of capturing tumor cells as seen with injected irradiated tumor cells, growing melanoma tumors deposit tumor-derived antigens into B cell follicles in patients (54). The accumulation of fluorescent tumor-derived antigen in the follicular dendritic cells in the germinal centers was observed using B16F10 melanoma. Depletion of the SCS macrophages ablated tumor-derived antigen accumulation in the follicular dendritic cells, demonstrating the necessity for SCS macrophages in depositing tumor-derived antigens into the B cell follicle (54). However, a recent publication has shown a contrasting observation; depletion of SCS macrophages increases tumor-derived exosome penetration deep into the B cell follicles and enhances B cell activation as measured by plasma immunoglobulin levels (15). In this study, growing tumors appear to disrupt the SCS macrophages in the tumor-draining LN and permits tumor-derived exosome entry into the B cell follicle. The increase in B cell response was correlated with a larger tumor size, suggesting the SCS macrophages are necessary to limit a pro-tumor B cell response (15). Because of these contradicting studies, further studies are required to reveal the function of SCS macrophage in anti-tumor immunity in the tumor-draining LN.

In contrast to the disruption of the macrophage layer seen in microbial infection, inflammation, or melanoma mentioned above, a recent study showed that inflammatory bowel disease increases the CD11b^+^CD169^+^ macrophages in the draining mesenteric LN. Depletion of the CD169^+^ macrophages in a CD169-DTR model showed reduced symptoms of inflammation, indicating that these macrophages promoted inflammation in the inflammatory bowel disease model (55). Whether these changes depend on the anatomical location of the LN or the disease models remains to be investigated.

## Conclusion and Prospective

The lymphatic system collects invading bacterial and viral pathogens and drains them to the LN for efficient processing and clearance. In this process, the LN sinus macrophages are among the first immune cells that interact with lymph-borne pathogens. With the evidence from different models, it is clear that SCS macrophages are essential for the response against lymph-borne pathogens. Unlike typical macrophages, the SCS macrophages are incapable of breaking down pathogens. The SCS macrophages appear to diversify its ability to target and initiate specific immune responses to a variety of lymph-borne pathogens by relaying antigens to B cells, producing cytokine signaling cascades to cause influx of dendritic cells, neutrophils, NK cells, or in some conditions, presenting antigens to T cells. Using cytokine production and immune cell recruitment, SCS macrophages can mount an early immune response against free-floating pathogens and prevent their LN invasion or systemic spreading. While there is a consensus that SCS macrophages limit the systemic dissemination of pathogens, there does not appear to be a universal mechanism for their action (Table 1). The requirement of SCS macrophages appear to be more critical for innate immunity, since depletion of SCS macrophages allow pathogens to escape the draining LN and spread systemically, adversely affecting survival rate. Surprisingly, although SCS macrophages appears critical to relay antigens to B cells, artificial depletion of SCS macrophages did not substantially interrupt the overall anti-microbial adaptive immune responses, except several hours of delay in the induction of adaptive immunity.

**Table 1:** Summary of SCS macrophages in different studies.

**Model**	**Cytokines**	**Recruited cells**	**SCS macrophages**	**Depletion method**	**References**
**VIRUSES**					
VSV	IFN-α, IFN-I	B cells, Plasmacytoid dendritic cells	–	CLL CD11c-DTR	(11, 33, 34)
Adenovirus	–	B cells	–	CLL	(11)
MCMV	–	–	–	CLL CD169-DTR	(35)
Influenza virus	IL-1α IFN-β	B cells NK cells Neutrophils	*MS DCs*, *MS macrophages* Necrosis	CLL CD169-DTR	(39, 40)
CpG	–	Dendritic Cells B cells	Dissociation, CCR7 dependent migration	CLL	(50)
**BACTERIA**					
*Pseudomonas aeruginosa*	IFN-γ IL-18 IL-1β	NK cells γδ T cells NKT cells αβ TCR CD8+ T cells Neutrophils	–	CLL	(36)
*Staphylococcus aureus*	C5aR	Neutrophils	Dissociation	CLL	(47, 50)
Lipid antigens (α-galactosylceramide coated on silica particles)	CD1d IL-2 IFN-γ	*i*NKT cells	Dissociation	CLL	(37, 50)
Glycolipids (α-linked galacturonic glycosphingolipid on silica particles)	–	*i*NKT cells	–	CLL	(37)
LPS	–	Dendritic Cells B cells	Dissociation, CCR7 dependent migration	CLL	(50)
**PARASITES**					
*Toxoplasma gondii*	IFN-γ	NK cells	Dissociation	CLL	(45, 49)
QS-21 (Malariacomponent)	IL-1β	Monocytes Neutrophils Eosinophils Dendritic cells		CLL	(46)
**CANCER**					
Exosomes	–	CD4+ T cells CD8+ T cells	–	CD169^−/−^	(38, 56)
Irradiated tumor cells	IFN-γ	CD8+ T cells		CD169-DTR	(12)
Melanoma and melanoma-derived exosomes	–	Follicular DCs B cells	Dissociation	CLL CD169-DTR	(15, 54)
**OTHER DISEASES**					
Colitis	IL-17, IL-21, IL-23, IL-6, IL-1β, TNFα, IL-12, IL-18, CCL8, CCL3	Th17 cells	Increase	CD169-DTR	(55)

Several studies have shown infection induces SCS macrophage dissociation from the SCS (Figure 2). CpG or LPS induces SCS macrophage migration deeper into the LN parenchyma, which impairs B cell responses to a secondary infection (50). Tumor progression induces SCS macrophage dissociation from the SCS in the tumor draining lymph node and results in B cell activation and tumor growth (15). Why the effect of dissociated SCS macrophages on subsequent immune protection appears contradictory between infectious diseases and cancer progression remains unclear. As it is now clear that inactivated influenza virus causes macrophage necrosis, one interpretation could be that challenge of microbes or materials mimicking microbial products causes more severe damage to SCS macrophages when compared to tumor derived antigens. Another possibility is that microbial product challenge lasts several hours, while infection or tumors may continuously deliver antigens for several days. Additionally, disease induced SCS macrophage dissociation also differs from the artificial SCS macrophage depletion as the former did not complete abrogate the SCS macrophage layer and some of these macrophages are relocated deeper into the LN parenchyma. Most studies use CLL and/or diphtheria toxin (DT) in a CD169-DTR to deplete SCS macrophages (Table 1) and both may cause off-target cell death. Additionally, the induced cell death may impact the function of immune cells in the LN. Thus, the mechanisms that cause SCS macrophage dissociation would substantially impact the immune protection to a subsequent challenge, such as secondary infection or continuous tumor-derived antigen delivery. More studies are required to understand why the SCS macrophages leave their position after stimulation and what is the immunological consequence of SCS macrophage dissociation from the SCS.

Currently, the mechanisms of how SCS macrophages participate in fighting against lymph-borne pathogens are better studied. The role of SCS macrophages in anti-tumor immunity in the tumor draining LN is still young. The collective literature in anti-microbial studies suggest future studies center around how SCS macrophage communication with other immune cells at different stages of tumor progression could provide pivotal insights into the development of immunotherapy.

## Author Contributions

All authors listed have made a substantial, direct and intellectual contribution to the work, and approved it for publication.

### Conflict of Interest Statement

The authors declare that the research was conducted in the absence of any commercial or financial relationships that could be construed as a potential conflict of interest.
